# Prevalence and risk factors of lower urinary tract symptoms in Chinese adult men: a multicentre cross-sectional study

**DOI:** 10.18632/oncotarget.22378

**Published:** 2017-11-06

**Authors:** Meng Rao, Huangfang Shangguan, Zhengyan Zeng, Yi Zheng, Huiping Zhang, Honggang Li, Wei Xia, Changhong Zhu, Chengliang Xiong, Huangtao Guan

**Affiliations:** ^1^ Family Planning Research Institute, Tongji Medical College, Huazhong University of Science and Technology, Wuhan, China; ^2^ College of Informatics, Huazhong Agricultural University, Wuhan, China; ^3^ Department of Neurology, The First Affiliated Hospital of Kunming Medical University, Kunming, China; ^4^ Department of Venereology, Wuhan Institute of Dermatology and Venereology, Wuhan, China; ^5^ Reproductive Medicine Center, Tongji Medical College, Huazhong University of Science and Technology, Wuhan, China

**Keywords:** LUTS, prevalence, risk factor, Chinese men, cross-sectional study

## Abstract

There has been no previous population-based study reporting the prevalence and risk factors of male lower urinary tract symptoms (LUTS) among men in mainland China. This cross-sectional study was conducted from 2013 to 2014 in three representative provinces of China: Guangdong, Hubei and Jiangsu. 3250 individuals participated in the interviews, which involved a questionnaire covering sociodemographic characteristics, lifestyle, dietary patterns and the International Prostate Symptom Score (IPSS). Blood was collected for lipids, glucose, insulin and reproductive hormone tests. The incidences of LUTS and its obstructive and irritative symptoms were calculated. Risk factors for LUTS were identified using multivariable logistic regression analysis. The prevalence of moderate to severe LUTS and its obstructive and irritative symptoms was 14.3%, 13.1% and 16.1%, respectively, and increased with age. The prevalence in Guangdong was much lower than that in Hubei and Jiangsu in different ages. Increased fasting plasma glucose and decreased HDL-C levels were associated with an increased risk of moderate to severe LUTS (OR = 1.30, 95% CI: 1.02–1.65 and OR = 2.06, 95% CI: 1.08–3.94, respectively). Free testosterone < 0.22 ng/ml decreased the risk of moderate to severe LUTS and obstructive and irritative symptoms by about 20–30%. An inadequate daily intake of vegetables, fruit and water significantly increased the risk of LUTS by 1.3–to 2.0 times. In conclusion, the prevalence of LUTS in Chinese men is high and increases with age. Dietary patterns may be critical for the development of LUTS. Thus, dietary modifications could be a useful strategy for preventing the development of LUTS.

## INTRODUCTION

Lower urinary tract symptoms (LUTS) is one of the most common clinical complaints in elderly men and has a significant effect on their quality of life [1]. LUTS is a cluster of chronic urinary symptoms in the bladder, prostate or urethra, and a major cause of benign prostatic hyperplasia (BPH) [2]. Population-based studies on the prevalence of LUTS have been conducted in the United States, Europe and Asia, in countries such as Korea, Singapore and Malaysia [3–10]. However, there has been no population-based study of LUTS in mainland China, except for two investigations conducted in Hong Kong. [11, 12]

While the identification of risk factors is important for the prevention of LUTS, apart from advancing age, there have been inconsistent conclusions about what may constitute a risk. Some studies have reported alcohol and cigarette consumption as an important risk factor for LUTS [6, 13]. Other investigations found that education and socio-economic status were linked to the incidence of LUTS [6, 14]. Metabolic syndrome has also been closely related to the incidence of LUTS [4, 6]. These inconsistent conclusions could be explained by differences in the populations under study. Therefore, population-specific risk factors are furthermore important for the understanding and prevention of LUTS.

The objective of this population-based study was thus to investigate the prevalence of LUTS in three representative provinces of China. We also aimed to evaluate the risk factors involved in the development of LUTS and its obstructive and irritative symptoms.

## RESULTS

### Subject characteristics

**Figure 1:**
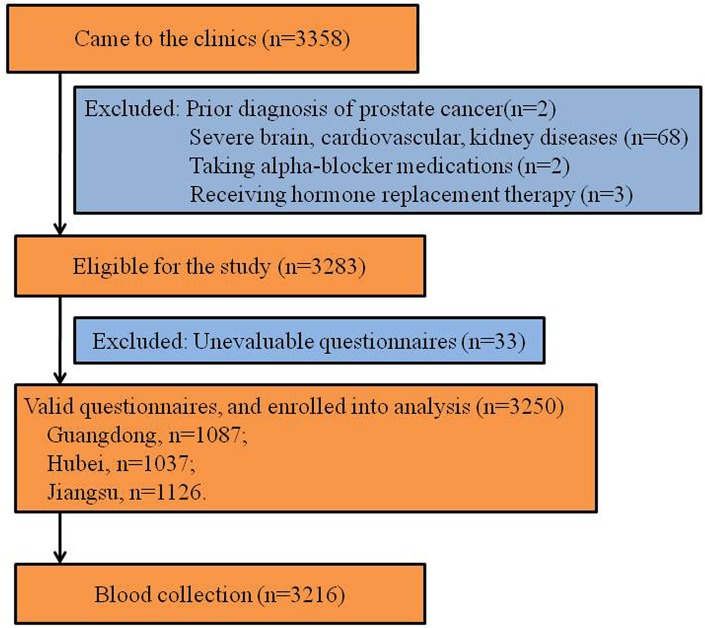
Study flowchart LUTS, lower urinary tract symptoms.

Out of the 3358 volunteers who came to the clinics, 3283 were eligible for the study based on the inclusion and exclusion criteria outlined above. All of these volunteers were asked to complete the questionnaires, but only 3250 were used in the analysis because the other 33 questionnaires had unintelligible or missing information. Of these, 1087 participants came from Guangdong, 1037 from Hubei and 1126 from Jiangsu (Figure 1), and 3216 participants completed the blood collection. The participants ranged from 18 to 82 years of age with an average age of 55.7 years. Sociodemographic characteristics, lifestyle, dietary habits and blood test results (lipids, FBG, HOMA-IR and testosterone) of the participants are shown in Table 1.

**Table 1:** Characteristics of sociodemographic, lifestyle, dietary patterns and serum parameters in Chinese men in different centers

Characteristics	Data				*p*
	Total	Guangdong *N* = 1087	Hubei *N* = 1037	Jiangsu *N* = 1126	
Mean age, year (SD)	55.7 (10.2)	55.4 (11.3)	55.2 (10.0)	56.5 (9.0)	0.007
Age, *n* (%)					
≤ 40	141 (4.3)	87	36	18	< 0.001
41–50	939 (28.9)	291	339	309	
51–5 60	1104 (33.9)	335	346	423	
61–5 70	818 (25.2)	274	235	309	
> 70	248 (7.6)	101	80	67	
Mean BMI, kg/m^2^ (SD)	24.4 (4.2)	23.8 (3.7)	23.8 (4.4)	25.5 (4.2)	< 0.001
BMI, *n* (%)					
< 18.5	112 (3.4)	46	52	14	
18.5–5 24.9	1867 (57.4)	691	641	535	
25–5 29.9	1072 (33.0)	280	291	501	
≥ 30	199 (6.2)	64	49	86	< 0.001
Education, *n* (%)					
≥ 9 years	1342 (41.3)	495	377	470	
< 9 years	1908 (58.7)	592	660	656	< 0.001
Residence, *n* (%)					
Rural	2461 (75.8)	1059	793	609	
Urban	789 (24.2)	28	244	515	< 0.001
Smoking, *n* (%) a					
Never	1216 (38.1)	470	332	414	
Former	379 (11.9)	108	127	144	
< 20 cigarette/day	938 (29.4)	193	328	417	
≥ 20 cigarettes/day	661 (20.7)	288	233	140	< 0.001
Alcohol drinking, *n* (%) b					
Never	876 (27.9)	316	279	281	
Former	159 (5.1)	44	62	53	
< 1 kg/week	1500 (47.7)	341	531	628	
≥ 1 kg/week	609 (19.4)	334	135	140	< 0.001
Hypertension, *n* (%) c					
No	1886 (59.5)	485	647	732	
Yes	1285 (40.5)	509	361	388	< 0.001
Vegetables intake, *n* (%)					
> 300 g/d	1239 (38.1)	638	253	348	
200–5 300 g/d	1033 (31.8)	245	401	387	
100–5 200 g/d	808 (24.9)	199	290	319	
< 100 g/d	170 (5.2)	5	93	72	< 0.001
Fruits intake, *n* (%)					
> 200 g/d	296 (9.1)	148	78	70	
100–5 200 g/d	872 (26.8)	555	154	163	
0–5 100 g/d	1353 (41.6)	365	496	492	
Almost no	729 (22.4)	19	309	401	<0.001
Water intake, *n* (%)					
> 1500 ml/d	1140 (35.1)	547	303	290	
1000–5 1500 ml/d	698 (21.5)	262	233	203	
500–5 1000 ml/d	1096 (33.7)	269	405	422	
< 500 ml/d	316 (9.7)	9	96	211	< 0.001
FBG, mmol/L, mean (SD)	5.76 (1.90)	5.66 (2.04)	5.12 (1.45)	6.45 (1.90)	< 0.001
HOMA-IR, mean (SD)	1.78 (3.71)	N/A	1.78 (3.71)	N/A	< 0.001
HDL-C, mmol/L, mean (SD)	1.74 (0.54)	1.84 (0.58)	1.53 (0.47)	1.83 (0.52)	< 0.001
TC, mmol/L, mean (SD)	5.64 (1.20)	5.97 (1.21)	5.53 (1.17)	5.43 (1.14)	< 0.001
TG, mmol/L, mean (SD)	1.90 (1.86)	1.90 (1.85)	1.67 (1.62)	2.11 (2.05)	< 0.001
TT, nmol/L, mean (SD)	16.56 (7.68)	17.82 (10.80)	17.85 (5.73)	14.20 (4.28)	< 0.001
FT, nmol/L, mean (SD)	0.26 (0.09)	0.25 (0.09)	0.27 (0.10)	0.25 (0.07)	< 0.001

^a^Cigarette smoking data from 56 subjects were missing; ^b^alcohol consumption data from 106 subjects were missing. ^c^Hypertension data from 79 subjects were missing; SD, standard deviation; N/A, not applicable. Chi-square test was used for the data analysis.

### Prevalence of LUTS

**Figure 2:**
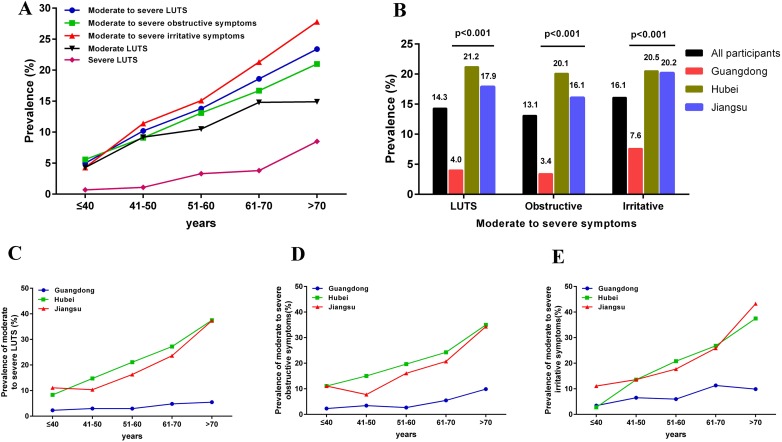
Prevalence of lower urinary tract symptoms (LUTS) of all participants and participants from different areas (**A**) prevalence of LUTS and its conponents in all participants across different ages; (**B**) prevalence of LUTS and its conponents in different areas; (**C**, **D** and **E**) represents the prevalence of LUTS and its conponents in different areas across different ages.

Overall, moderate to severe LUTS and obstructive and irritative symptoms were reported by 14.3%, 13.1% and 16.1% of the participants, respectively. The prevalence of these three symptoms all increased with age. For instance, the incidences of moderate to severe LUTS, obstructive and irritative symptoms were 5.0%, 5.6% and 4.3%, respectively, for subjects less than 40 years of age, whereas these proportions increased to 23.4%, 21.0% and 27.8%, respectively, for subjects older than 70 years. Additionally, the participants in Guangdong experienced a much lower prevalence than those in Hubei and Jiangsu across all age groups. The prevalence of moderate to severe LUTS for men aged over 70 years were 5.5%, 37.5% and 37.5% in Guangdong, Hubei and Jiangsu, respectively. For obstructive symptoms, the percentages were 9.9%, 35.0% and 34.3%, and for irritative symptoms they were 9.9%, 37.5% and 43.3%, respectively (Figure 2).

### Subject characteristics among different LUTS groups

A total of 2785 subjects reported having mild LUTS (IPSS < 7), 366 subjects were diagnosed with moderate LUTS (IPSS 8–19) and 99 with severe LUTS (IPSS > 19). Age and BMI were significantly different among the groups (*p* < 0.001 and 0.03, respectively). For serum lipid levels, the HDL-C from subjects with moderate LUTS (1.7 mmol/l) was lower than subjects with mild (1.8 mmol/l) and severe LUTS (1.8 mmol/l) (*p* = 0.005). Nevertheless, the TC levels in subjects with severe LUTS (5.4 mmol/l) were significantly lower than for subjects with mild (5.7 mmol/l) and severe (5.5 mmol/l) LUTS (*p* = 0.008). As for testosterone levels, TT, but not FT, was higher in severe LUTS subjects (18.2 nmol/l) than in mild (16.4 nmol/l) and moderate (17.1 nmol/l) LUTS subjects (*p* = 0.03) (Table 2).

**Table 2:** Demographic and clinical parameters in different LUTS groups [mean (SD)]

Parameters	Mild LUTS	Moderate LUTS	Severe LUTS	*p*
	(IPSS 0–7)	(IPSS 8–19)	(IPSS 20–35)	
	*N* = 2785	*N* = 366	*N* = 99	
Age	55.2 (10.1)	58.1 (9.6)	61.8 (8.7)	< 0.0001
BMI (Kg/m^2^)	24.4 (4.1)	24.7 (4.9 )	23.5 (2.9)	0.03
SBP (mmHg)	135.7 (21.2)	135.6 (20.5)	136.9 (24.1)	0.85
DBP (mmHg)	85.0 (12.1)	84.8 (12.0)	85.8 (12.8)	0.76
FBG (mmol/L)	5.7 (1.9)	5.9 (1.8)	5.9 (1.6)	0.33
HOMA-IR	1.7 (3.8)	2.2 (3.6)	1.4 (1.7)	0.31
HDL-C (mmol/L)	1.8 (0.5)	1.7 (0.5)	1.8 (0.5)	0.005
TC (mmol/L)	5.7 (1.2)	5.5 (1.2)	5.4 (1.2)	0.008
TG (mmol/L)	1.9 (1.9)	1.8 (1.9)	1.6 (1.2)	0.17
TT (nmol/L)	16.4 (7.5)	17.1 (7.4)	18.2 (11.8)	0.03
FT (nmol/L)	0.26 (0.09)	0.26 (0.09)	0.24 (0.08)	0.07

SD, Standard deviation; SBP, Systolic blood pressure; DBP, Diastolic blood pressure; FBG, Fasting plasma glucose; HOMA-IR, homeostasis model assessment, insulin resistence; TC, total cholesterol; TG, triglycerides; TT, total testosterone; FT, free testosterone, the same below.

A one-way ANOVA was used for the data analysis.

### Correlation of the IPSS and its obstructive and irritative components with sociodemographic and clinical parameters (controlling for age)

Both age and FBG were positively correlated with IPSS (*r* = 0.166, *p* < 0.0001 and *r* = 0.038, *p* = 0.033, respectively), whereas HDL-C and TC were negatively related to IPSS (*r* = -0.089, *p* < 0.001 and *r* = -0.06, *p* = 0.001, respectively). The results also showed a positive correlation between age, BMI, FBG and QoL (all *p* < 0.01), and a negative correlation between SBP, HDL-C, TC and QoL (all *p* < 0.01). Age was positively associated with obstructive and irritative symptoms (*r* = 0.125, *p* < 0.001 and *r* = 0.196, *p* < 0.001, respectively), whereas HDL-C and TC were negatively correlated with both obstructive and irritative symptoms (all *p* < 0.01). Additionally, there was a positive relationship between HOMA-IR and irritative symptoms (*r* = 0.079, *p* = 0.013), and between FT levels and obstructive symptoms (*r* = 0.038, *p* = 0.03) (Table 3).

**Table 3:** Correlation between different parameters and the IPSS

Parameters	IPSS		QoL		Obstructive		irritative	
	*r*	*p*	*r*	*p*	*r*	*p*	*r*	*p*
Age	0.166	<.0001	0.109	<.0001	0.125	<.0001	0.196	<.0001
BMI (Kg/m^2^)	0.03	0.14	0.05	0.01	0.02	0.37	0.04	0.045
SBP (mmHg)	-0.04	0.047	-0.06	< 0.001	-0.04	0.03	-0.03	0.16
DBP (mmHg)	0.005	0.78	0.016	0.38	0.002	0.92	0.009	0.62
FBG (mmol/L)	0.038	0.033	0.048	0.006	0.01	0.56	0.07	< 0.001
HOMA-IR	0.061	0.055	0.019	0.553	0.044	0.171	0.079	0.013
HDL-C (mmol/L)	-0.089	< 0.001	-0.097	< 0.001	-0.074	< 0.001	-0.094	< 0.001
TC (mmol/L)	-0.06	0.001	-0.06	< 0.001	-0.056	0.002	-0.055	0.002
TG (mmol/L)	-0.015	0.405	0.018	0.299	-0.02	0.259	-0.005	0.786
TT (nmol/L)	0.032	0.073	-0.024	0.17	0.033	0.064	0.025	0.163
FT (nmol/L)	0.032	0.069	0.024	0.182	0.038	0.03	0.018	0.310

The abbreviations are the same with above.

IPSS, International Prostate Symptom Score; QoL, quality of life;

Obstructive symptoms, the questions No. 1, 3, 5 and 6 of the IPSS are categorized as the assessment of obstructive symptoms

Irritative symptoms, the questions No. 2, 4, and 7 of the IPSS are categorized as the evaluation of irritative symptoms.

### Potential risk factors for LUTS

According to logistic regression analysis, the risk factors for LUTS are shown in Table 4 (age was adjusted). Increased age was one of the most obvious risk factors for LUTS, QoL and obstructive and irritative symptoms: there was a 5.8-fold, 3.9-fold, 4.4-fold and 8.7-fold increase, respectively, for men aged older than 70 years compared with men younger than 40 years. Daily alcohol consumption less than 1000 g increased the risk of moderate to severe LUTS and the obstructive symptoms, whereas consumption greater than 1000 g decreased the risk of all types of LUTS by about 40–50%, taking never drinkers as a reference. Hyperglycemia was positively associated with the risk of moderate to severe LUTS (OR = 1.26, 95% CI: 1.01–1.57), whereas decreased levels of HDL-C were positively related to the QoL scores and obstructive symptoms (OR = 1.70, 95% CI: 1.06–2.73 and OR = 2.04, 95% CI: 1.14–3.66, respectively). Increased TC levels decreased the risk of all of the LUTS indices. FT level was positively correlated with the risk for moderate to severe LUTS and its components. Furthermore, the risks of all these LUTS indices increased with the decrease in the daily intake of vegetables, fruit and water (Table 4). After the adjustment for all the related confounders, except for research centre and TT levels (Table 5), advancing age was still the most important risk factor for all LUTS indices. Men aged over 70 years experienced a much higher increase in the incidence of moderate to severe LUTS symptoms (OR = 7.77, 95% CI: 3.08, 19.60), QoL (OR = 3.74, 95% CI: 2.12, 6.61), obstructive symptoms (OR = 5.44, 95% CI: 2.25, 13.17) and irritative symptoms (OR = 8.65, 95% CI: 3.47, 21.55) compared with men aged less than 40 years. Heavy drinking (> 1000 g/week) was still associated with decreased QoL scores and obstructive symptoms. Hyperglycemia increased the risk of moderate to severe LUTS (OR = 1.30, 95% CI). Dyslipidemia, including increased levels of TC and TG and decreased levels of HDL-C, affected the obstructive symptoms and the QoL. Lower level of FT decreased the risk of moderate to severe LUTS, obstructive and irritative symptoms by about 20–30%. As for dietary habits, the daily intake of vegetables was negatively associated with the incidence of all types of LUTS, whereas daily water intake was only negatively related with the QoL and incidence of moderate to severe LUTS. Nevertheless, only men who ate almost no fruit experienced a 1.7- to 2.0 times increased risk of moderate to severe LUTS (OR = 1.67, 95% CI, 1.05, 2.65) and obstructive symptoms (OR = 1.98, 95% CI, 1.20, 3.27), respectively, compared with men who ate more than 200 g of fruit per day.

**Table 4:** Association between participant characteristics and LUTS was analyzed using logistic regression adjusting for age [OR (95% CI)]

Characteristics	Moderate to severe LUTS	QoL (> 2)	Moderate to severe obstructive symptoms	Moderate to severe irritative symptoms
Age				
≤ 40	Ref.	Ref.	Ref.	Ref.
41–50	2.18 (0.99, 4.80)	1.82 (1.16, 2.88)	1.66 (0.78, 3.49)	2.89 (1.25, 6.72)
51–60	3.06 (1.40, 6.67)	2.79 (1.78, 4.37)	2.52 (1.21, 5.25)	4.01 (1.74, 9.25)
61–70	4.37 (2.00, 9.53)	3.25 (2.06, 5.12)	3.32 (1.59, 6.93)	6.08 (2.64, 14.01)
> 70	5.84 (2.59, 13.20)	3.88 (2.36, 6.40)	4.41 (2.03, 9.59)	8.67 (3.66, 20.57)
BMI				
< 18.5	0.86 (0.56, 1.31)	0.80 (0.53, 1.21)	105 (0.61, 1.81)	0.78 (0.46, 1.34)
18.5–24.9	Ref.	Ref.	Ref.	Ref.
25–29.9	0.75 (0.38, 1.46)	1.19 (1.01, 1.39)	1.08 (0.86, 1.36)	1.06 (0.86, 1.31)
≥ 30	0.96 (0.62, 1.48)	1.11 (0.81, 1.52)	1.17 (0.75, 1.81)	1.24 (0.83, 1.86)
Center				
Guangdong	Ref.	Ref.	Ref.	Ref.
Hubei	6.74 (4.80, 9.47)	3.45 (2.82, 4.22)	7.52 (5.22, 10.83)	3.25 (2.47, 4.28)
Jiangsu	5.24 (3.72, 7.37)	4.19 (3.44, 5.11)	5.53 (3.83, 7.99)	3.10 (2.37, 4.07)
Education				
< 9 years	Ref.	Ref.	Ref.	Ref.
≥ 9 years	1.20 (0.95, 1.51)	1.03 (0.93, 1.34)	1.26 (0.99, 1.60)	1.08 (0.87, 1.34)
Residence				
Urban	Ref.	Ref.	Ref.	Ref.
Rural	0.93 (0.74, 1.16)	1.19 (1.01, 1.44)	0.86 (0.68, 1.08)	0.88 (0.70, 1.10)
Cigarette smoking				
Never	Ref.	Ref.	Ref.	Ref.
Former	1.16 (0.84, 1.59)	1.26 (0.99, 1.60)	1.32 (0.95, 1.82)	1.08 (0.80, 1.47)
< 20 cigarettes/d	1.15 (0.90, 1.46)	1.14 (0.96, 1.37)	1.32 (1.02, 1.69)	1.08 (0.86, 1.36)
≥ 20 cigarettes/d	0.75 (0.56, 1.01)	0.87 (0.71, 1.07)	0.85 (0.62, 1.15)	0.72 (0.54, 0.95)
Alcohol drinking				
Never	Ref.	Ref.	Ref.	Ref.
Former	1.24 (0.80, 1.92)	1.20 (0.85, 1.69)	1.28 (0.82, 2.01)	1.72 (1.16, 2.55)
< 1000 g/week	1.30 (1.03, 1.65)	1.16 (0.97, 1.39)	1.33 (1.04, 1.70)	1.26 (1.00, 1.58)
≥ 1000 g/week	0.55 (0.39, 0.78)	0.58 (0.46, 0.73)	0.49 (0.34, 0.72)	0.61 (0.44, 0.84)
Hypertension				
No	Ref.	Ref.	Ref.	Ref.
Yes	0.90 (0.73, 1.11)	0.94 (0.81, 1.09)	0.86 (0.69, 1.06)	0.97 (0.80, 1.18)
FBG (mmol/L)				
≤ 6.1	ref.	Ref.	Ref.	Ref.
> 6.1	1.26 (1.01, 1.57)	1.12 (0.95, 1.32)	1.17 (0.93, 1.47)	1.23 (1.00, 1.52)
HDL-C (mmol/L)				
< 0.9	1.68 (0.93, 3.06)	1.70 (1.06, 2.73)	2.04 (1.14, 3.66)	0.97 (0.49, 1.92)
≥ 0.9	Ref.	Ref.	Ref.	Ref.
TC (mmol/L)				
≤ 5.7	Ref.	Ref.	Ref.	Ref.
> 5.7	0.78 (0.64, 0.96)	0.83 (0.71, 0.96)	0.70 (0.56, 0.87)	0.78 (0.65, 0.96)
TG (mmol/L)				
≤ 1.7	Ref.	Ref.	Ref.	Ref.
> 1.7	0.89 (0.71, 1.10)	1.07 (0.92, 1.25)	0.80 (0.64, 1.00)	0.99 (0.81, 1.21)
TT (nmol/L)				
< 11	0.91 (0.69, 1.20)	1.24 (1.02, 1.51)	0.91 (0.69, 1.21)	0.97 (0.75, 1.26)
≥ 11	Ref.	Ref.	Ref.	Ref.
FT (nmol/L)				
< 0.22	0.71 (0.57, 0.90)	0.93 (0.79, 1.10)	0.76 (0.60, 0.97)	0.74 (0.60, 0.92)
≥ 0.22	Ref.	Ref.	Ref.	Ref.
Vegetables intake				
> 300 g/d	Ref.	Ref.	Ref.	Ref.
200–300 g/d	1.55 (1.22, 1.99)	2.21 (1.85, 2.65)	1.72 (1.33, 2.22)	1.45 (1.15, 1.83)
100–200 g/d	1.77 (1.36, 2.29)	2.48 (2.04, 3.01)	1.86 (1.42, 2.45)	1.51 (1.17, 1.93)
< 100 g/d	1.93 (1.24, 3.01)	3.01 (2.15, 4.21)	2.39 (1.53, 3.73)	1.92 (1.26, 2.91)
Fruits intake				
> 200 g/d	Ref.	Ref.	Ref.	Ref.
100–200 g/d	0.84 (0.54, 1.32)	1.01 (0.75, 1.35)	0.94 (0.57, 1.52)	0.71 (0.47, 1.06)
0–100 g/d	1.64 (1.09, 2.48)	1.55 (1.17, 2.05)	1.78 (1.14, 2.79)	1.23 (0.85, 1.77)
Almost none	2.40 (1.57, 3.67)	2.19 (1.63, 2.94)	2.81 (1.78, 4.44)	1.92 (1.31, 2.80)
Water intake				
> 1500 ml/d	Ref.	Ref.	Ref.	Ref.
1000–1500 ml/d	1.44 (1.08, 1.92)	1.50 (1.23, 1.84)	1.49 (1.11, 2.00)	1.29 (0.99, 1.68)
500–1000 ml/d	1.73 (1.34, 2.22)	1.27 (1.06, 1.52)	1.65 (1.27, 2.14)	1.44 (1.13, 1.82)
< 500 ml/d	2.33 (1.67, 3.25)	2.19 (1.70, 2.84)	2.10 (1.48, 2.97)	2.03 (1.48, 2.79)

LUTS, lower urinary tract symptoms; Ref. reference; OR, odds ratio; CI, confidence interval.

**Table 5:** Association between participant characteristics and LUTS was analyzed using logistic regression adjusting for all the parameters except for research center and TT [OR (9% CI)]

Characteristics	Moderate to severe LUTS	QoL (>2)	Moderate to severe obstructive symptoms	Moderate to severe irritative symptoms
Age				
≤ 40	Ref.	Ref.	Ref.	Ref.
41–50	2.19 (0.93, 5.17)	1.56 (0.96, 2.53)	1.61 (0.72, 3.62)	2.42 (1.03, 5.68)
51–60	3.34 (1.43, 7.84)	2.68 (1.66, 4.34)	2.71 (1.22, 6.03)	3.56 (1.52, 8.32)
61–70	5.51 (2.31, 13.15)	3.26 (1.97, 5.37)	4.07 (1.79, 9.24)	5.85 (2.47, 13.86)
> 70	7.77 (3.08, 19.60)	3.74 (2.12, 6.61)	5.44 (2.25, 13.17)	8.65 (3.47, 21.55)
BMI				
< 18.5	0.77 (0.42, 1.44)	0.79 (0.51, 1.25)	0.91 (0.50, 1.65)	0.72 (0.40, 1.31)
18.5–24.9	Ref.	Ref.	Ref.	Ref.
25–29.9	1.05 (0.82, 1.34)	1.13 (0.94, 1.36)	1.03 (0.80, 1.32)	0.99 (0.79, 1.25)
≥ 30	1.19 (0.75, 1.91)	1.14 (0.80, 1.62)	1.19 (0.74, 1.94)	1.20 (0.77, 1.87)
Education				
< 9 years	Ref.	Ref.	Ref.	Ref.
≥9 years	1.02 (0.80, 1.30)	0.89 (0.74, 1.07)	1.07 (0.83, 1.38)	0.91 (0.73, 1.15)
Residence a				
Urban	Ref.	Ref.	Ref.	Ref.
Rural	0.95 (0.75, 1.22)	1.17 (0.96, 1.43)	0.87 (0.68, 1.12)	0.86 (0.68, 1.10)
Cigarette smoking				
Never	Ref.	Ref.	Ref.	Ref.
Former	1.01 (0.71, 1.42)	1.10 (0.84, 1.43)	1.13 (0.79, 1.62)	0.88 (0.63, 1.23)
< 20 cigarettes/d	0.99 (0.77, 1.29)	0.96 (0.79, 1.17)	1.10 (0.84, 1.44)	0.95 (0.74, 1.22)
≥ 20 cigarettes/d	0.82 (0.60, 1.12)	0.94 (0.75, 1.18)	0.91 (0.66, 1.27)	0.75 (0.56, 1.02)
Alcohol drinking				
Never	Ref.	Ref.	Ref.	Ref.
Former	1.25 (0.77, 2.02)	1.04 (0.71, 1.53)	1.18 (0.72, 1.94)	1.85 (1.19, 2.86)
< 1000 g/week	1.28 (1.00, 1.66)	1.06 (0.87, 1.29)	1.27 (0.98, 1.65)	1.28 (1.00, 1.64)
≥ 1000 g/week	0.70 (0.48, 1.02)	0.64 (0.49, 0.82)	0.61 (0.41, 0.90)	0.77 (0.55, 1.09)
Hypertension				
No	Ref.	Ref.	Ref.	Ref.
Yes	1.00 (0.80, 1.24)	1.06 (0.90, 1.26)	0.99 (0.79, 1.25)	1.07 (0.87, 1.33)
FBG (mmol/L)				
≤ 6.1	ref.	Ref.	Ref.	Ref.
> 6.1	1.30 (1.02, 165)	1.03 (0.85, 1.24)	1.25 (0.97, 1.61)	1.18 (0.93, 1.49)
HDL-C (mmol/L)				
#x003C; 0.9	2.06 (1.08, 3.94)	1.51 (0.88, 2.59)	2.42 (1.26, 4.65)	1.08 (0.53, 2.22)
≥ 0.9	Ref.	Ref.	Ref.	Ref.
TC (mmol/L)				
≤ 5.7	Ref.	Ref.	Ref.	Ref.
> 5.7	0.84 (0.67, 1.05)	0.86 (0.73, 1.02)	0.78 (0.62, 0.99)	0.86 (0.69, 1.06)
TG (mmol/L)				
≤ 1.7	Ref.	Ref.	Ref.	Ref.
> 1.7	0.81 (0.63, 1.05)	1.06 (0.88, 1.27)	0.74 (0.56, 0.96)	0.99 (0.78, 1.25)
FT (nmol/L)				
< 0.22	0.73 (0.58, 0.93)	0.95 (0.80, 1.14)	0.77 (0.60, 0.99)	0.78 (0.62, 0.97)
≥ 0.22	Ref.	Ref.	Ref.	Ref.
Vegetables intake				
> 300 g/d	Ref.	Ref.	Ref.	Ref.
200–300 g/d	1.35 (1.04, 1.77)	2.04 (1.67, 2.48)	1.48 (1.12, 1.95)	1.33 (1.03, 1.71)
100–200 g/d	1.44 (1.08, 1.92)	2.34 (1.88, 2.90)	1.55 (1.15, 2.10)	1.31 (1.00, 1.73)
< 100 g/d	1.42 (0.89, 2.28)	2.40 (1.66, 3.47)	1.79 (1.11, 2.89)	1.55 (0.99, 2.43)
Fruits intake				
> 200 g/d	Ref.	Ref.	Ref.	Ref.
100–200 g/d	0.83 (0.52, 1.34)	0.97 (0.70, 1.35)	0.94 (0.56, 1.57)	0.71 (0.46, 1.08)
0–100 g/d	1.32 (0.85, 2.07)	1.31 (0.95, 1.79)	1.46 (0.90, 2.38)	1.04 (0.70, 1.55)
Almost no	1.67 (1.05, 2.65)	1.64 (1.18, 2.29)	1.98 (1.20, 3.27)	1.45 (0.96, 2.20)
Water intake				
> 1500 ml/d	Ref.	Ref.	Ref.	Ref.
1000–1500 ml/d	1.27 (0.93, 1.73)	1.31 (1.05, 1.64)	1.28 (0.93, 1.75)	1.13 (0.85, 1.51)
500–1000 ml/d	1.37 (1.04, 1.80)	0.97 (0.79, 1.19)	1.26 (0.95, 1.68)	1.16 (0.89, 1.50)
< 500 ml/d	1.51 (1.05, 2.18)	1.40 (1.05, 1.86)	1.26 (0.86, 1.85)	1.40 (0.99, 1.98)

LUTS, lower urinary tract symptoms; Ref. reference; OR, odds ratio; CI, confidence interval.

^a^For residence, only participants from Hubei and Jiangsu were enrolled into the analysis since almost all participants from Guangdong lived in rural areas.

All parameters except research center and TT were adjusted in this multivariable logistic regression model.

## DISCUSSION

This is the first multicentre study aimed at investigating the prevalence and risk factors of LUTS in Chinese men. We conducted a PubMed search for English-language articles focused on the prevalence of LUTS in men. These studies also used IPSS to evaluate the risk of LUTS and took total IPSS scores > 7 to indicate moderate to severe LUTS. Similar to other studies, our data showed an increase in the prevalence of moderate to severe LUTS with advancing age. The prevalence of LUTS in our study was much lower compared with other studies based in Europe [3–5, 7, 14], the US [6], South America [18], Hong Kong [12] and other Asian countries [3, 10], especially among people aged over 50 years. Nevertheless, a similar prevalence was found between this study and another 2 population-based studies in Norway [8] and Singapore [9], for men who were younger than 50 and 70 years, respectively. This difference in the prevalence may be partially due to the different study designs (face-to-face interviews or self-completed questionnaires), population characteristics such as their jobs or areas, and people's ethnicity and life habits [3, 10]

A total of 141 men aged between 18–40 years were enrolled in this study as a control, since men aged over 40 years were found to suffered from a much higher risk of LUTS in a series of studies [8, 14]. The overall prevalence of moderate to severe LUTS and obstructive and irritative symptoms varied significantly among the three areas in our study. Men aged over 70 years in Hubei and Jiangsu experienced a 7-fold higher risk for moderate to severe LUTS than men in Guangdong. It is worth noting that men aged over 70 in Guangdong, Hubei and Jiangsu were 101, 80 and 67, respectively. The numbers were not as much as those in other age groups, whereas it was still obvious that prevalence of LUTS in Guangdong was much higher than other two areas. The critical reason for this difference may be the participants’ dietary patterns, as the daily intake of vegetables, fruit and water differed significantly between these three areas. As shown in Table 1, almost 60% of subjects in Guangdong had a daily vegetable intake of more than 300 g, whereas in Hubei and Jiangsu the proportion was 24% and 31%, respectively. Our multi-logistic regression analysis showed that a vegetable intake of less than 300 g/day could increase the risk of LUTS by almost 1.4 times, 2–2.4 times for QoL and 1.3–1.8 times for obstructive and irritative symptoms. This is consistent with a population-based study in Hong Kong, which also concluded that adequate fruit and vegetable intake was associated with improved LUTS among Chinese elderly men [12] Another Prostate Cancer Prevention Trial in the US reported that high consumption of vegetables resulted in a lower risk of developing a BPH, the principal underlying cause of LUTS [19]. People with almost no fruit intake experienced a 1.7 times higher risk of LUTS compared with those who consumed enough fruit (300 g/day), even though fruit intake was not so closely related to the irritative symptoms. This result was supported by another two case control studies [20, 21] and a population-based cross-sectional study [22], which indicated an inverse correlation of vegetable and fruit intake and BPH risk. Nevertheless, in a population-based cohort study among Japanese-American men there was no association observed for vegetable and fruit juice consumption (6581 men, including 846 cases) [23] Rohrmann et al. investigated the association of fruit and vegetable consumption and the intake of micronutrients with BPH in a large US cohort study (32265 men, including 6092 cases). They found that a higher intake of vegetables, but not fruit or fruit juice, could decrease the risk of BPH [24] Further analysis, however, indicated that consumption of fruit rich in β-carotene, lutein and vitamin C was associated with a lower incidence of BPH. Differences in the populations, data collection methods and enrolment of confounding factors when performing data analysis could explain the inconsistent conclusions among these studies. In general, our results suggested a risk factor of daily inadequate vegetable and fruit intake for LUTS. One potential explanation for this phenomenon could be that vegetables and fruit contain high levels of antioxidant properties, such as vitamin E and lycopene, which are critical for attenuating oxidative damage, as evidenced by studies where oxidative damage was associated with the development of BPH [25, 26]. Second, dietary factors may alter endogenous sex hormone metabolism in men [27] and effect the sympathetic nervous system [28]. It is possible that vegetable and fruit intake could moderate both the hormonally regulated prostate growth and heightened smooth muscle tone that cause BPH [29]. Additionally, some vegetables and fruits contain vitamin E, a lipid-soluble Vitamin which is easily absorbed by fat. Fruits and vegetables play a very important role in regulating intestinal function, which can reduce constipation and reduce congestion of the prostate. This may be another important reasons in improving LUTS with adequate vegetable and fruit intake. It is also worth noting that an inadequate daily water intake (< 1000 ml/day) significantly increased the incidence of moderate to severe LUTS and QoL, but not obstructive and irritative symptoms. This may be due to the flushing effect that water has on the lower urinary tract, because urethritis is also an important reason for LUTS. Overall, physicians and the public should pay attention to dietary modifications that can improve prostate health due to their highly effective, noninvasive and modifiable properties.

While several studies have linked the association between LUTS and metabolic symptoms and its components, including obesity, abnormal glucose and lipid metabolism, and hypertension [4, 6, 30–33], no consensus has been reached until now. We found that FBG, but not HOMA-IR, was positively correlated with IPSS, QoL and irritative symptoms, and FBG > 6.1 nmol/l was associated with an increased risk of moderate to severe LUTS. This was consistent with another population-based study in France, which also reported a positive relationship between FBG levels and the severity of LUTS [4]. Another two studies in Norway and the US indicated that diabetes increased the risk of moderate to severe LUTS, by 1.3- and 2.0 times, respectively [6, 8]. Nevertheless, in contrast to our results, Gao et al. conducted a study with 3103 Chinese men and showed that FBG was not associated with the risk of LUTS [33]. As for lipid metabolism, we found that decreased HDL-C was associated with an increased risk of moderate to severe LUTS. This was in accordance with several previously published studies, which also reported an association between the incidence or severity of LUTS and serum levels of HDL-C [34–36]. Some other investigators, however, failed to establish a link between dyslipidemia and LUTS [13, 33].

It is widely accepted that prostate growth is dependent on the presence of androgens and that a gradual increase in prostate volume reflects the development of BPH. Our results showed a lower risk of moderate to severe LUTS and irritative symptoms for those subjects whose FT levels were less than 0.22 nmol/l, the threshold commonly used for the diagnosis of late-onset hypogonadism (LOH). However, it remains a controversial question whether testosterone replacement therapy for the treatment of LOH effects LUTS [37, 38]. Further clinical studies are thus needed to draw a convincing conclusion. There are conflicting data regarding the effect of cigarette and alcohol consumption on the risk of LUTS. Joseph et al., for instance, demonstrated that both cigarette and alcohol consumption increased the incidence of LUTS. [6] Other studies, in contrast, found no effect or even a protective effect of cigarette or alcohol consumption on the development of LUTS [39–41]. Our results showed no obvious association between cigarette consumption and LUTS, but a protective effect of heavy alcohol consumption (> 1000 g/week) on LUTS. This may be due to the increased serum estrogen and decreased testosterone levels resulting from acute and repeated alcohol consumption [6, 42]. Additionally, some early drinkers may stop drinking due to moderate to severe LUTS, while the individuals who are not suffered from LUTS will keep on drinking. This may be an important bias and may be evidenced by the prospective study in the future.

This study has several strengths. First, as a multicentre study, a large sample was selected from three representative areas. Second, the same group of well-trained urologists collected the data with good consistency. Additionally, we included a series of confounders, including demography, life habits, dietary patterns and some important serum parameters to draw a reliable conclusion. This study also has several limitations. On one hand, as a cross-sectional study, it was not possible to ascertain risk factors, and longitudinal studies are needed to better understand the development of LUTS. On the other hand, the diet questionnaire did not collect information on the detailed nutrients, which would be useful to further investigate the effect of dietary patterns on LUTS.

In conclusion, Chinese adult men also have a high prevalence of LUTS and the prevalence varies significantly in different areas, although the prevalence is much lower than that in most other countries. Our results underline age as the strongest risk factor for LUTS. Increased FBG and decreased HDL-C levels were associated with an increased risk of LUTS. We also found evidence that dietary patterns, including inadequate daily intake of vegetables, fruit and water, increased the risk of LUTS. Although further studies are needed to confirm the results, it is possible that dietary modification could be useful for preventing the development of LUTS.

## MATERIALS AND METHODS

### Study design

This project was supported by the 12th Five-Year Plan of National Science and Technology of China (2012BAI32B03) and is part of the project “Reproductive health survey of Chinese middle-aged and elderly men.” The study proposal was approved by the Ethics Committee of Tongji Medical College, Huazhong University of Science and Technology (No. 20130311), and conducted according to their guidelines for research. All participants provided their full written informed consent.

The study involved a cross-sectional multicentre survey carried out from March 2013 to May 2014, as described in our previous study [15]. Three provinces of Guangdong (southern China), Hubei (central China), Jiangsu (eastern China) were selected for this study, and a total of 12 districts with a mix of urban and rural locations were arranged for subjects recruitment based on the characteristics of their geography, economic development and population density. Our research group collaborated with the local health and family planning commission to recruit volunteers. Posters were distributed to each family and subjects who came to the local family planning clinics were recruited if they met the minimum eligibility criteria for the study.

### Study population

The subjects who participated in the study were required to meet the following inclusion criteria: 1) aged over 18 years old, 2) normally developed and 3) could communicate with the researchers and complete the questionnaires. The exclusion criteria included: 1) severe brain, cardiovascular, or reproductive endocrine disorders, kidney disease or prostatitis, 2) previous diagnosis of prostate cancer, 3) hormone replacement therapy in the past 3 months or 4) recent rectal examination, prostate biopsy or cystoscopy. All the subjects were required to complete a routine examination, questionnaire and blood collection.

### Data collection

The routine examination involved height, weight and blood pressure measurements. Body Mass Index (BMI) was calculated by dividing weight (kg) by the square of height (m). Blood pressure, including systolic blood pressure (SBP) and diastolic blood pressure (DBP), were measured in the sitting position after a 5-min rest by using a mercury sphygmomanometer. SBP ≥ 140 mmHg and/or DBP ≥ 90 mmHg indicated hypertension. Hypertension was also diagnosed in the participants based on any previous diagnosis by doctors. The questionnaire involved a series of detailed questions, covering age, residence (urban or rural), education, history of hypertension, diabetes mellitus, dyslipidemia and treatment, history of cigarette and alcohol consumption, dietary habits and the the International Prostate Symptom Score (IPSS). Smoking status was classified into three groups: former (at least 100 cigarettes smoked in a lifetime), current and never smoking. Current smoking was further categorised into ≤ 20 and > 20 cigarettes per day based on the number of cigarettes consumed daily during the past year. Drinking status was similarly defined as former, current (at least 12 drinks in the previous year) or never drinking. For current drinkers, the estimation of usual weekly alcohol consumption was calculated by combining the intake of beer and wine together. Approximately, 12 bottles of beer was equivalent to 0.5 kg of alcohol. Current drinking was further categorised into ≤ 1 and > 1 kg/week based on the alcohol intake amount in the past year. For dietary habits, we mainly focused on the daily intake of vegetables, fruit and water. To obtain the information conveniently and reliably, we used standard containers as references to reflect the daily intake of vegetables, fruit and water. The participants were classified into different groups according to their estimated average daily intake of vegetables, fruit and water in the past year: vegetables: < 100 g, 100–200 g, 200–300 g and > 300 g per day; fruit :almost none, < 100 g, 100–200 g and > 200 g per day; and water: < 500 ml, 500–1000 ml, 1000–1500 ml and > 1500 ml per day.

### The assessment of LUTS

The IPSS is an internationally recognised valid method for the evaluation of LUTS and based on the answers to seven questions, concerned with the feeling of incomplete bladder emptying, frequency, intermittency, urgency, weak stream, straining and nocturia. The answers for each question are assigned points from 0 to 5. The total score can therefore range from 0 to 35 (1–7, mild; 8–19, moderate; 20–35, severe). It also contains a question about the quality of life (Qol), which is assigned a score from 1 to 6. The QoL scores were defined as mild (0–2) or moderate to severe (2–6). The questions 1, 3, 5 and 6 could also be categorised as the assessment of obstructive symptoms (≥ 5 out of 20 points was considered to indicate the presence of moderate to severe obstructive symptoms). The questions 2, 4 and 7 were categorised as the evaluation of irritative symptoms (≥ 4 out of 15 points was considered to indicate the presence of moderate to severe irritative symptoms) [6, 13].

### Blood collection and hormone/lipids/glucose/insulin assay

After completing the questionnaire, venous blood samples were collected from the participants between 7:00–10:00 am after overnight fasting. The samples were stored at each centre temporarily and were all tested in the reproductive centre of Tongji Medical College. Serum total testosterone (TT) and sex hormone-binding globulin (SHBG) were tested using a chemiluminescent immunoassay method on a UniCel DxI 800 analyser (Beckman Coulter, Brea, USA). Free testosterone (FT) concentration was calculated by the Vermeulen formula [16]. The serum concentrations of fasting plasma glucose (FBG), insulin, HDL-C, total cholesterol (TC) and triglyceride (TG) were measured directly with a cobas c 311 system (Roche Diagnostic system) (only samples collected from Hubei were tested for the insulin concentration). A homeostasis model assessment (HOMA) index was calculated according to the formula (FBG [mmol/L] × fasting insulin [mU/L])/22.5 [17]. The HOMA index was used to evaluate the levels of insulin resistance HOMA-IR.

### Statistical analysis

Sociodemographic characteristics, lifestyle, dietary patterns and the prevalence of moderate to severe LUTS, QoL and obstructive and irritative symptoms in each centre were compared using Chi-square tests. The average age, BMI and serum parameters in each centre were compared with the analysis of one-way ANOVA. The participants were categorised into three groups of mild, moderate and severe LUTS according to their IPSS scores. A one-way ANOVA was performed to evaluate the difference of age, BMI, blood pressure and serum parameters across the ordered categories. The correlation of the IPSS, QoL and obstructive and irritative components with selected parameters was assessed using Pearson's partial correlation (controlling for the age of the subjects). Additionally, two multi-variable logistic regression models were used to assess the association between sociodemographic characteristics, lifestyle, dietary patterns and the risk of moderate to severe LUTS, QoL and the obstructive and irritative symptoms. First, we only adjusted for age because it was the main factor affecting the development of LUTS. Then, we adjusted all of the parameters except for research centre and TT, because the research centre had a collinearity with age and TT was not as important as FT. A two-sided *p* value < 0.05 was considered for statistical significance. Data analysis was performed using SPSS 17.0 (SPSS Inc., Chicago, IL, USA).

## Abbreviations

BMI: body mass index
BPH: benign prostatic hyperplasia
DBP: diastolic blood pressure
FBG: fasting plasma glucose
FT: Free testosterone
HOMA-IR: homeostasis model assessment- insulin resistance
HDL-C: high-density lipoprotein-cholesterol
IPSS: International Prostate Symptom Score
LUTS: lower urinary tract symptoms
QoL: quality of life
SBP: systolic blood pressure
TT: total testosterone
TC: total cholesterol
TG: triglyceride
